# Effect of genetic ancestry to the risk of susceptibility to gastric cancer in a mixed population of the Brazilian Amazon

**DOI:** 10.1186/s13104-017-2963-4

**Published:** 2017-11-29

**Authors:** Ellen Moreno da Silva, Marianne Fernandes Rodrigues, Darlen Cardoso de Carvalho, Luciana Pereira Colares Leitao, Giovanna Chaves Cavalcante, Esdras Edgar Batista Pereira, Antônio André Conde Modesto, João Farias Guerreiro, Paulo Pimentel de Assumpção, Sidney Emanuel Batista dos Santos, Ney Pereira Carneiro dos Santos

**Affiliations:** 1 Núcleo de Pesquisas em Oncologia, Hospital Universitário João de Barros Barreto, Universidade Federal do Pará, Belém, Brazil; 2 Laboratório de Genética Humana e Médica, Universidade Federal do Pará, Belém, PA Brazil; 3 Hospital Universitário João de Barros Barreto, Universidade Federal do Pará, Belém, PA Brazil; 4 Instituto de Ciências Biológicas, Laboratório de Genética Humana e Médica, Cidade Universitária Prof. José da Silveira Netto, Rua Augusto Corrêa, 01, BOX: 8615, Belém, PA CEP 66.075-970 Brazil

## Abstract

**Background:**

Global literature describes differences in the incidence of gastric cancer among populations. For instance, Europeans have lower incidence rates of gastric cancer in relation to Latin and Asian populations, particularly Korean and Japanese populations. However, only a few studies have been able to verify the occurrence of gastric cancer in admixed populations with high interethnic degree mix, such as the Brazilian Amazon region.

**Results:**

We observed an increase in European ancestry in the control group compared to the case group (47% vs. 41%). Using increments of 10%, compared to categorical distribution of European ancestry in the sample, we found a difference in the contribution between cases and controls (p = 0.03). Multiple logistic regression was performed to determine the influence of European ancestry in susceptibility to gastric cancer in the sample. According to the adopted model, for each 10% increase in European ancestry, there is a 20% decrease chance of developing gastric cancer (P = 0.0121; OR = 0.81; 95% CI 0.54–0.83).

**Conclusion:**

Overall, the results suggest that a greater contribution of European ancestry can be a protective factor for the development of gastric cancer in the studied Amazon population. It can help to establish protocols able to predict susceptibility to gastric cancer in admixed populations.

**Electronic supplementary material:**

The online version of this article (10.1186/s13104-017-2963-4) contains supplementary material, which is available to authorized users.

## Background

Stomach cancer, also known as gastric cancer, is the third leading cause of cancer death worldwide [1]. The incidence of stomach cancer is the sixth highest in the world [2] and is among the highest incidences in Latin America [3].

The literature reports differences in the incidence of gastric cancer among populations. For example, Europeans have lower rates of gastric cancer incidence when compared to Latin and Asian populations, especially Japanese and Korean [4, 5]. These differences in incidence can be attributed to risk factors associated with lifestyle, such as diet, obesity, stress and physical inactivity. In addition, other studies suggest that the lower incidence of gastric cancer in the European population may be related to lower frequency of genes associated with gastric cancer susceptibility [5, 6].

In this context, it is conceivable that heterogeneity among populations and different genetic ancestries can contribute to the varying levels of susceptibility to cancer development. This is especially important in admixed populations, such as the Brazilian population, which is known to be one of the most heterogeneous populations in the world, with contributions from three main parental groups: Amerindian, European and African [7–9]. Literature demonstrates the influence of genetic ancestry and the risk of developing various types of cancer, such as breast [10–12], colorectal [13], leukemia [14] and ovarian cancer [15]. More specifically, there is a work that explores the influence of genetic ancestry in the risk of developing gastric cancer among Brazilian populations [12].

Therefore, the objective of this study is to investigate the influence of genetic ancestry in susceptibility to gastric cancer in a population of the Amazon region with a high degree of interethnic admixture.

## Methods

### Cases and control

The participants of the research were chosen based on a case–control study. Participants from both case and control groups were recruited from free services in public institutions, were from the same socioeconomic level, and belonged to the same geographic area.

The case group constituted of 137 individuals that were diagnosed with gastric adenocarcinoma, attended by the Unified Health System (SUS), in a public hospital that is reference in the treatment of this kind of neoplasm (Hospital Universitário João de Barros Barreto, Pará, Brazil). Most patients involved in the study (80%) presented advanced tumor staging—grade III and IV according to Borrmann classification.

The control group constituted of 262 cancer-free individuals, attended in the Laboratório de Exercício Resistido e de Saúde (LERES), from a public university of the region (Universidade Estadual do Pará, Pará, Brazil).

### Ethical approval

The protocol used in the study was approved by the Ethics Committee of the University Hospital João de Barros Barreto (Protocol Number 3505/2004). All patients in the present study signed a consent form.

### Analysis of genetic ancestry

The analysis of genetic ancestry was performed using a panel of 48 ancestry informative markers (AIM) developed by Santos et al. [16]. Amplification was performed using three multiplex PCR reactions with 16 markers each. PCR separation and analysis was performed by capillary electrophoresis using the ABI PRISM 3130 sequencing and GeneMapper ID Software v3.2. Individual proportions of ancestries of Europeans, Africans and Amerindians were estimated using the STRUCTURE software v2.3.3, with three parental populations (European, African and Amerindian).

Parental populations involved individuals that were representative of three great ancestral groups: 222 Amerindian from nine tribes in the Brazilian Amazon (Tiriyó, Waiãpi, Zoé, Urubu-Kaapor, Awa-Guajá, Parakanã, Wai Wai, Gavião and Zoró), 211 African (Angola, Mozambique, Congo Republic, Cameroon and Ivory Coast) and 268 European (Portugal and Spain). More details on these populations can be found in [17].

### Statistical analysis

All statistical analyses were performed using the statistical program SPSS v.20.0 (SPSS, Chicago, IL. USA). Chi squared test in pairs was applied for group comparisons of categorical variables (sex), while Student’s t-test was used for the analysis of quantitative variables (age). For comparisons of ancestry among the samples, we used the Mann–Whitney test. Multiple logistic regression analyses were performed to estimate odds ratios (ORs) and 95% confidence intervals (CIs). In these analyses, variables considered confounding factors (age, sex) were corrected. All statistical tests were two-tailed and based on P < 0.05 probability to be significant.

## Results

We analyzed 137 patients with gastric cancer and 262 cancer-free individuals. Table 1 shows the demographic characteristics of these groups. The case group showed a predominance of men while the control group showed a predominance of women. The results were statistically significant between the case and control groups regarding the following variables: age (P < 0.001), sex (P < 0.001) and European ancestry (P = 0.001).

**Table 1:** Demographic variables for patients with gastric cancer and the control group

Variable	Case	Control	*P*
Age, years^a^	52.81 ± 18.89	46.80 ± 24.48	< 0.001
Sex (women/men)	34/103	156/106	< 0.001
Genetic ancestry^b^			
European ancestry	0.415 ± 0.143	0.466 ± 0.158	0.001
African ancestry	0.259 ± 0.140	0.232 ± 0.117	0.089
Amerindian ancestry	0.326 ± 0.142	0.302 ± 0.137	0.079

^a^Values are as expressed as mean (± SD = standard deviation). Significance determined by Student’s *t*-test

^b^Values are as expressed as mean ± SD. Significance determined by Mann–Whitney test

According to the ancestry analyses conducted in this study, the ethnic composition of the case group was 41% European, 26% African and 33% Amerindian. By comparison, the control group’s ancestry contributions was 47% European, 23% African and 30% Amerindian (Table 1). The results revealed that there was a higher contribution of European ancestry in the control group compared to the case group (47% vs. 41%). Using increments of 10%, we compared the categorical distribution of European ancestry in the sample and found a difference in this ancestry contribution between cases and controls (P = 0.03) (Table 2).

**Table 2:** Categorical distribution of European ancestry in patients with gastric cancer in the comparison with the control group

Genetic ancestry (%)	Case no. (%)	Control no. (%)
European ancestry		
10–20	8 (6)	14 (5.3)
20–30	24 (17.5)	32 (12.2)
30–40	30 (21.8)	40 (15.2)
40–50	38 (27.7)	60 (23)
50–60	21 (15.3)	61 (23.2)
> 60	16 (11.6)	55 (21)
*P*	0.03	

Multiple logistic regressions were performed to determine the influence of European ancestry in the susceptibility to gastric cancer in the sample (Table 3). According to the adopted model, for each 10% increase in European ancestry there is a 20% lower chance of developing gastric cancer (P = 0.0121; OR = 0.81; 95% CI 0.68–0.95).

**Table 3:** Odds ratio (OR) and 95% confidence intervals (CIs) in the logistic regression model of European ancestry

Genetic ancestry	Case mean	Control mean	OR^a^	95% CI^a^	*P* ^a^
European ancestry	0.415 ± 0.143	0.466 ± 0.158	0.807	0.682–0.953	0.0121

^a^Logistic regression adjusted for sex and age. Odds-ratio of the analysis of 10% ethnicity increase

## Discussion

In our analyses, the case group presented a greater proportion of men (75%), while in the control group, the proportion was greater among women (60%). The incidence of gastric cancer, as well as other gastrointestinal neoplasms, was greater in men than in women. This finding has been observed in different populations worldwide [18–22]. The justification for this disparity in incidence between men and women goes beyond the explanation of sex differences for exposure to known risk factors. Many studies have suggested a protective effect of estrogen in the development of gastric cancer [22, 23].

To better comprehend the sex difference in the susceptibility to gastric cancer, we performed new statistical tests, which included isolated analyses for men (Additional file 1) and for women (Additional file 2). In both men and women, the European ancestry was the most prevalent, being more frequent in the control group than in the case group.

In the analyses involving only men (Additional file 1), European ancestry was statistically different between case and control (P = 0.009). Logistic regression analysis showed a reduction of 94% in the chances of developing gastric cancer (P = 0.004; OR = 0.059; 95% CI 0.008–0.414).

In the analyses involving only women (Additional file 2), there was no statistically significant difference of European ancestry between case and control groups (P = 0.052). We believe that the absence of significance may be due to the low sample number of women in the case group (N = 34), which may not allow reliable statistical associations.

**Fig. 1:**
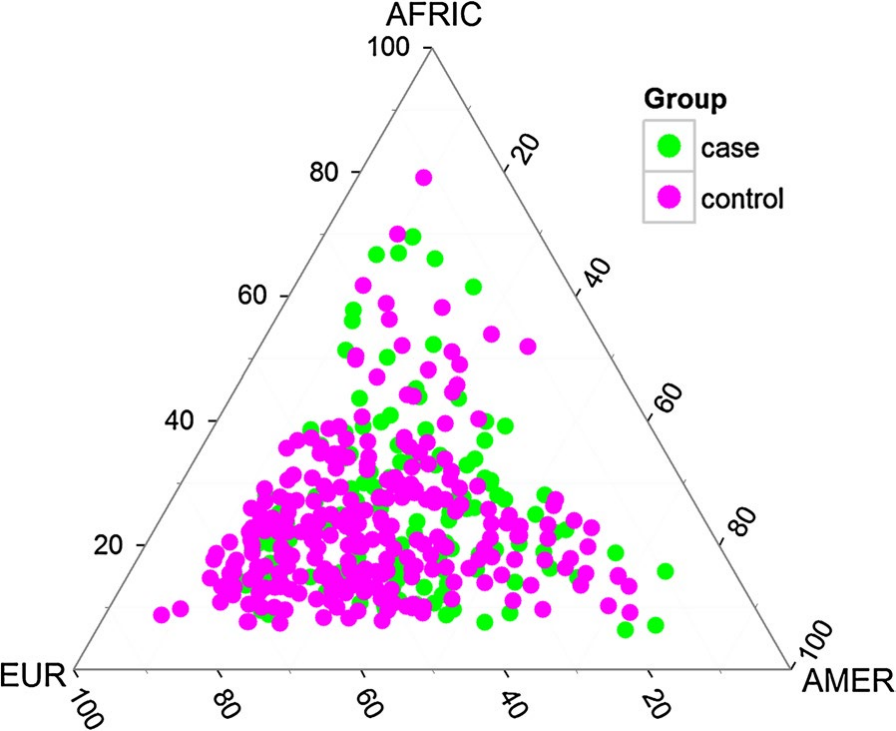
Individual interethnic mixture representation. Cancer patients are represented by points in green and control subjects by dots in purple, their locations in the graph correspond to the mixing ratio. The mixture is estimated by comparison with populations of parent individuals represented in the vertices of the triangle: European, Amerindian and African

Brazilian population is one of the most heterogeneous populations worldwide and it is formed by an admixture of Amerindians, Europeans and Africans. The admixture process occurred through different means in the Brazilian geographic regions. Our sample presented a variety in its composition, with a higher prevalence of European ancestry, followed by Amerindian and African ancestries in both case and control groups (Fig. 1). Previous data have demonstrated that the European ancestry is predominant in four regions in Brazil—North (68.8%), Northeast (60.1%), Southeast (74.2%) and South (79.5%). The African proportion was highest in the Northeast (30.3%) and the Amerindian proportion was highest in the North (19.4%) [16, 24].

Our results demonstrated that European ancestry is more represented in the control group than it is in the case group (Table 1) and that genetic ancestry modifies the risk of developing gastric cancer. The 10% increase in European ancestry was associated with decreased risk of developing gastric cancer (Table 3). There are only a few works in literature on the association of genetic ancestry and gastric cancer susceptibility [12, 25].

The work by [12] has investigated the susceptibility to gastric and breast cancer together, in a different population from the North region of Brazil, and found a protection association between European ancestry and the development of these neoplasms. Each increase of 10% in the European ancestry was inversely correlated to the risk of developing cancer (OR = 0.186), corroborating with our results.

A work developed by [25] has investigated the contribution of genetic ancestry in the risk of developing gastric cancer in an admixed population from Lima (Peru). European ancestry was correlated to a negative effect in the development of gastric cancer, supporting our results.

In addition, other studies have analyzed the association of genetic ancestry and cancers around the world. For example, African ancestry was associated with colorectal cancer [13] and with prostate cancer [26]. Amerindian ancestry was associated with the development of acute lymphoblastic leukemia (ALL) [27].

Our results showed that genetic ancestry may modify the risk of developing gastric cancer. European ancestry was related to a reduction in the chances of developing this neoplasm. Further studies must be carried out to identify genetic polymorphisms that are more frequent in populations with high European ancestry and that may confer a protective effect to this ancestry regarding the development of gastric cancer.

## Conclusion

Our results corroborate with studies that suggest that the lower incidence of gastric cancer in the European population may be related to lower frequency of alleles related to high susceptibility of developing gastric cancer [5, 6]. Therefore, a higher European ancestry contribution may be considered a protection factor to gastric cancer in the studied Amazon population. More studies are needed to confirm such results, but this work presented significant findings that contribute to a greater knowledge of the influence of genetic ancestry in the development of gastric cancer.

## Additional files

**Additional file 1.** Demographic variables for men in both groups (gastric cancer and control). To better comprehend the sex difference in the susceptibility to gastric cancer, we performed new statistical tests, which included isolated analyses for men.

**Additional file 2.** Demographic variables for women in both groups (gastric cancer and control). To better comprehend the sex difference in the susceptibility to gastric cancer, we performed new statistical tests, which included isolated analyses for women.

## Abbreviations

ALL: acute lymphoblastic leukemia
PCR: polymerase chain reaction
OR: odds-ratio
