# Correction to: Effect of genetic ancestry to the risk of susceptibility to gastric cancer in a mixed population of the Brazilian Amazon

**DOI:** 10.1186/s13104-017-3075-x

**Published:** 2017-12-21

**Authors:** Ellen Moreno da Silva, Marianne Rodrigues Fernandes, Darlen Cardoso de Carvalho, Luciana Pereira Colares Leitao, Giovanna Chaves Cavalcante, Esdras Edgar Batista Pereira, Antônio André Conde Modesto, João Farias Guerreiro, Paulo Pimentel de Assumpção, Sidney Emanuel Batista dos Santos, Ney Pereira Carneiro dos Santos

**Affiliations:** 1 Núcleo de Pesquisas em Oncologia, Hospital Universitário João de Barros Barreto, Universidade Federal do Pará, Belém, Brazil; 2 Laboratório de Genética Humana e Médica, Universidade Federal do Pará, Belém, PA Brazil; 3 Hospital Universitário João de Barros Barreto, Universidade Federal do Pará, Belém, PA Brazil; 4 Instituto de Ciências Biológicas, Laboratório de Genética Humana e Médica, Cidade Universitária Prof. José da Silveira Netto, Rua Augusto Corrêa, 01, BOX: 8615, Belém, PA CEP 66.075-970 Brazil

## Correction to: BMC Res Notes (2017) 10:646 10.1186/s13104-017-2963-4

Following publication of the original article [1], the authors requested a correction to the name of one of the co-authors. The correct name is Marianne Rodrigues Fernandes, not Marianne Fernandes Rodrigues.

The original article has been updated.
