# Supplementary material for: Effect of genetic ancestry to the risk of susceptibility to gastric cancer in a mixed population of the Brazilian Amazon
Source: BMC Res Notes. 2017 Nov 29;10:646. doi: 10.1186/s13104-017-2963-4 (PMC5707813; doi:10.1186/s13104-017-2963-4)
Supplement: Supplementary file 2 — Additional file 2. Demographic variables for women in both groups (gastric cancer and control). To better comprehend the sex difference in the susceptibility to gastric cancer, we performed new statistical tests, which included isolated analyses for women. [file 13104_2017_2963_MOESM2_ESM.docx]

**Additional File 2.** Demographic variables for women in both groups (gastric cancer and control).

| **Variable** | **Case** | **Control** | ***p-*Value** |
| --- | --- | --- | --- |
| **Women** |  |  |  |
| Age, years ^a^ | 53.75±12.82 | 47.96±24.59 | 0.261 |
| Genetic Ancestry ^b^ |  |  |  |
| European Ancestry^c^ | 0.410±0.135 | 0.462±0.155 | 0.052 |
| African Ancestry | 0.342±0.147 | 0.312±0.142 | 0.173 |
| Amerindian Ancestry | 0.248±0.151 | 0.224±0.119 | 0.788 |

^a^ Values are expressed as mean (±SD = standard deviation). Significance determined by Student’s *t*-test.

^b^ Values are expressed as mean ±SD. Significance determined by Mann-Whitney test.

^c^ Logistic regression adjusted for age: P=0.434; OR=0.318; 95%CI=0.018-5.628.
